# Mycobacterial infection of breast prosthesis – a conservative treatment: a case report

**DOI:** 10.1186/1471-2334-14-238

**Published:** 2014-05-05

**Authors:** David Atallah, Nadine El Kassis, George Araj, Marwan Nasr, Roy Nasnas, Nicolas Veziris, Dolla Sarkis

**Affiliations:** 1 Department of Gynecology and Obstetrics, Hôtel-Dieu de France University Hospital, Saint Joseph University, Beirut, Lebanon; 2 Department of Pathology and Laboratory Medicine, American University of Beirut Medical Center, Beirut, Lebanon; 3 Department of Plastic Surgery, Hôtel-Dieu de France University Hospital, Saint Joseph University, Beirut, Lebanon; 4 Department of Infectious Diseases, Hôtel-Dieu de France University Hospital, Saint Joseph University, Beirut, Lebanon; 5 Sorbonne University, Université Pierre et Marie Curie-Paris 6, CR7, Centre d’Immunologie et des Maladies Infectieuses (CIMI), team E13 (Bacteriology), 75013 Paris, France; 6 INSERM, U1135, Centre d’Immunologie et des Maladies Infectieuses (CIMI), team E13 (Bacteriology), 75013 Paris, France; 7 AP-HP, Hôpital Pitié-Salpêtrière, Centre National de Référence des Mycobactéries et de la Résistance des Mycobactéries aux Antituberculeux, Bactériologie-Hygiène, 75013 Paris, France; 8 Faculty of Pharmacy, Saint Joseph University, Beirut, Lebanon

**Keywords:** Antibiotherapy, Conservative treatment, Mastectomy, *Mycobacterium canariasense*, Prosthesis, Sparing

## Abstract

**Background:**

Bacterial infection is a well-known risk of breast implant surgery. It is typically caused by bacterial skin flora, specifically *Staphylococcus aureus* and the coagulase negative staphylococci. There have been infrequent reports of breast implant infection caused by the atypical mycobacteria, of which *Mycobacterium canariasense* not yet reported in the literature.

**Case presentation:**

This report summarizes the case of a female patient who underwent mastectomy followed by bilateral breast augmentation and presented approximately three years later with clinical evidence of infected breast prosthesis by *Mycobacterium canariasense*. One year after thoroughly follow-up, appropriate antibiotherapy and the change of the infected prosthesis, the patient presented no signs of reinfection.

**Conclusion:**

Our case demonstrates that *Mycobacterium canariasense* should be considered as a new potential cause of infected breast prosthesis.

## Background

Breast implant-associated bacterial infections occur in 2.0 to 2.5% of cosmetic cases and up to 20% of reconstructive cases [1]. Infections caused by mycobacteria are uncommon, but are being increasingly reported [2-5]. However, breast implant infections caused by *Mycobacterium canariasense* are atypical and not yet reported in the literature.

We report here the case of a female patient who underwent mastectomy followed by bilateral breast augmentation and presented approximately three years later with clinical evidence of infected breast prosthesis by *Mycobacterium canariasense*. The complexities established in the diagnosis and need for a thorough and accurate microbiologic evaluation, as well as the management strategies are summarized in the case report and discussion that ensue.

## Case presentation

A 44-year old healthy woman was operated on May 2006, of bilateral mastectomy for an invasive lobular carcinoma and associated in situ lobular carcinoma in the left breast. On the left side, a skin sparing radical modified mastectomy was performed, and on the right side a prophylactic nipple sparing simple mastectomy was done. Mastectomy was followed by bilateral breast augmentation with Mentor prosthesis (Mentor Worldwide LLC, California, USA) filled with 150 mL saline serum, the prosthesis was inserted behind the pectoral muscles.

Prophylactic antibiotherapy with amoxicillin and clavulanate 2 g/day was prescribed for 10 days. The patient received later on, chemotherapy and radiotherapy treatment. Two years later, she had her Mentor® prosthesis changed with a similar new one filled with 110 mL of gel and 215 cc of saline serum for unsatisfactory esthetic results. Valves were inserted on the lateral thoracic wall. Augmentin® (amoxicillin and clavulanate) was given intravenously 30 minutes before surgery and then was continued for 10 days.

As a follow-up, she was seeing her plastic surgeon on a regular basis and her prosthesis were progressively filled with saline serum through the valves. Her postoperative course was uneventful until January 2009, when she presented with edema and redness of her right breast after valve ablation, without pus drainage nor fever. She was treated with empiric antibiotherapy (Augmentin® 2 g/day and ciprofloxacin 500 mg twice daily) for 10 days and received local care. Her serum white blood cell count was 6,000 (neutrophils of 81%). Consequently, since she had no improvement of her symptoms, she underwent removal of her right breast implant with capsulectomy and massive irrigation with bacitracin and 9 liters of saline serum, then new prosthesis re-implantation.

Serous fluid drained from her right breast was sent to culture, as well as the capsule and the whole prosthesis. Gram stain, aerobic and anaerobic cultures of the fluid, prosthesis, and breast tissue returned negative. Histopathological examination of the breast capsule revealed none specific inflammatory changes without identifying any germ. Fungal culture returned negative as well after 40 days of incubation. Furthermore, acid-fast stain of the fluid drained from the right breast, debridement tissue, and the prosthesis returned negative. Acid-fast culture of the fluid, and of the resected right breast tissue were also negative, while acid-fast culture of the ablated prosthesis were positive, it revealed the presence of an acid-fast bacilli resembling to *Mycobacterium tuberculosis*. A swab culture of the periprosthetic space – the space between the prosthesis and the breast wall – as well as a definitive mycobacterial culture of the unidentified pathogen isolated from the prosthesis in the initial laboratory were sent to the National Reference Center on Mycobacteria of Pitié-Salpêtrière hospital in Paris, France. The antimicrobial susceptibility testing of the pathogen were obtained from Rapid Growing Mycobacteria Plate Format (RAPMYCO) Sensititre plates in Mueller-Hinton medium incubated for 7 days. The identification was done by sequencing the gene of the Heat Shock Protein (HSP) and revealed the presence of *M. canariasense* in the prosthesis and the peri-prosthetic space.

Subsequently, the patient received post-operatively empirical antibiotherapy including vancomycin 1 g every 12 hours, Tienam® (imipinem/cilastatin) 500 mg every 6 hours, and Tavanic® (levofloxacin) 500 mg/day, for 10 days. She was then discharged on oral tritherapy: ciprofloxacin, Bactrim® (sulfamethoxazole and trimethoprim) and clindamycin for 6 months, after the culture revealed the presence of an atypical *Mycobacterium.* The first two antibiotics were prescribed based on the antibiogram’s results (Table 1). However, clindamycin was prescribed by the infectious disease specialist without performing clindamycin susceptibility testing. The patient was last seen in March 2014. She was symptom-free, without signs of infection and achieved a satisfactory psychological, psychosocial and esthetic results.

**Table 1:** **Antimicrobial susceptibility of *Mycobacterium canariasense*- Antibiogram as performed in Paris, France**

**Antibiotic**	**Susceptibility**	**Minimum inhibitory concentrations (mg/L)**	**Pharmaceutical form (in case of pathogen’s sensitivity to the antibiotic)**
Amoxicillin + clavulanic acid	R	64.00	-
Cefoxitin	S	16.00	Powder for solution for injection, IV
Ceftriaxone	R	> 64.00	-
Imipenem	I	8.00	-
Streptomycin	S	2.00	Powder for solution for injection, IM/IV
Tobramycin	S	2.00	Inhalation powder
			Ophthalmic ointment and solution
			Powder for solution for perfusion
Amikacin	S	2.00	Powder for solution for injection
Minocycline	I	2.00	-
Tigecycline	S	0.02	Powder for solution for injection
Clarithromycin	S	1.00	Per os, not available in Lebanon
Cotrimoxazole (sulfamethoxazole + trimethoprim)	S	1.00	Per os, prescribed to the patient
			Powder for solution for injection or infusion
Ciprofloxacin	S	0.50	Ophthalmic ointment and solution
		1.00	Per os, prescribed to the patient
			Solution for auricular instillation
			Solution for perfusion, IV
Ethambutol	S	<0.50	Per os, not available in Lebanon
			Solution for injection
Rifabutine	R	1.00	-
Linezolide	S	4.00	Per os, not available in Lebanon
			Solution for perfusion

I: intermediate; IM: intramuscular; IV: intravenous; R: resistant; S: sensitive.

## Conclusions

Breast prostheses are being increasingly used, both for cosmetic and reconstructive purposes [3]. However, periprosthetic infection is perhaps the most feared and least understood complication of these procedures [3]. Although bacteria such as *Staphylococcus aureus*, coagulase-negative staphylococci and mycobacteria such as *Mycobacterium fortuitum* complex are the most common cause of surgical site infections [3,4,6], this case highlights the emergence of atypical and unusual *M. canariasense* as pathogen associated with breast implant infections. Our review of the literature has produced no similar breast implant infection.

*M. canariasense* is a member of the rapidly growing, non-pigmented and atypical mycobacterium group. The characterization of this mycobacterium, in 2004, was based on a cluster of strains isolated from blood cultures via indwelling catheters from patients with probable nosocomial infection in the Canary Islands, Spain [7,8]. It was also isolated from respiratory sources but the pulmonary infection was deemed possible to doubtful in a patient primary diagnosed with cancer [9].

Regarding management of breast implants’ infections, although prospective studies are lacking, published reports recommend that the mammary prosthesis be removed, with earlier removal favored to prevent implant extrusion and tissue contracture [10]. Besides, the best conservative treatment of the implant seems to be the association of implant change, irrigations and antibiotherapy [11]. These procedures were substantially performed in our case.

Although the cause of breast implant infections might not be truly understood, the challenge is how to treat them appropriately. Classic teaching holds that periprosthetic infection mandates implant removal and delayed reinsertion after the infection clears [12]. However, the first report of immediate salvage of infected breast prostheses after complete mastectomy for cancer was by Yii and Khoo in 2003 [13]. Similarly, a more recent study by Chun *et al.* revealed that eight (100%) patients had positive outcomes following the immediate salvage [12]. In the latter case series, the author reported that the cultures of the periprosthetic fluid of the infected implants were positive for *Staphylococcus aureus* in three patients and *Enterococcus faecalis* in one patient. A fifth patient was positive for *Staphylococcus epidermidis*[12]. In our case, given that the patient insisted to conserve her esthetic results, she was given the choice of delayed or immediate re-implantation. At that stage of time, the causal agent of the infection was not yet identified as a rapidly growing mycobacteria. In close, she decided to undergo the one-stage re-implantation although she was aware of the risk of infection recurrence. Consequently, early and aggressive surgical intervention resulted in successful immediate implant salvage. It is worthy to note that delayed re-implantation may have major psychological implications for the patient. To many women suffering with breast cancer, breast reconstruction is a critical component in the recovery process. Explantation halts the reconstruction process for upward of 6 months. In this context, one of the goals of the immediate re-implantation is to improve women’s psychological well-being.

On the other side, despite the continuous evolution of mycobacterial taxonomy which may represent a source of confusion for laboratories and clinicians, and which may not be identified by conventional procedures, we were able in our settings to identify the atypical *M. canariasense.* In fact, definitive diagnosis of mycobacterial breast implant infection requires demonstration of the organism from the periprosthetic site. Thus, specimens must be submitted for acid-fast stains fungal and mycobacterial culture, in addition to standard analyses, including Gram-stain, bacterial aerobic and anaerobic culture, and histopathology [3]. In this case, it was only after multiple specimens of the fluid drained from the right breast, debridement tissue, and the prosthesis were sterile on routine bacterial and fungal cultures, that the possibility of an atypical infection such as a mycobacterial process was considered. Subsequent specimens submitted for mycobacterial culture from the ablated prosthesis identified the causative pathogen, *M. canariasense*. Therefore, we prescribed a prolonged course of anti-mycobacterial therapy that allowed for eradication of the infection.

Moreover, empiric antimicrobial therapy was started in our case pending isolation and susceptibility testing results. Of note, in the study of Xiang et *al.*, the results of the antimicrobial susceptibility of *M. canariasense* showed that this agent is sensitive to amikacin, imipenem, ciprofloxacin and trimethoprim-sulfamethoxazole. It is intermediately susceptible to cefoxitin. However, it is resistant to clarithromycin and minocycline [9]. With this knowledge, our patient was discharged on ciprofloxacin, clindamycin and sulfamethoxazole-trimethoprim, after the culture revealed the presence of *M. canariasense*. We stress the point that clindamycin was empirically prescribed by the infectious disease specialist although nontuberculous mycobacteria (NTM) generally are not susceptible to this agent. He aimed to cover a large spectrum of infectious agents due to the non-conventional diagnosis and management of our patient. In the end, the treatment provided anti-bacterial coverage for the re-implanted prosthesis for 6 months.

In conclusion, the theme of this article is to share a successful one-stage replacement of infected breast prosthesis by atypical NTM after mastectomy reconstruction. The surgical and pharmacological management of the patient was successful given the very good esthetic results in the patient. Our case also reminds us that a high index of suspicion for unusual pathogens, such as the atypical mycobacteria, is necessary when considering infections that do not improve despite seemingly appropriate management. Moreover, it demonstrates that *M. canariasense* should be considered as a new potential cause of infected breast prosthesis.

## Consent

Written informed consent was obtained from the patient for publication of this case report. A copy of the written consent is available for review by the Editor-in-Chief of this journal.

## Abbreviations

HSP: Heat shock protein; I: Intermediate; IM: Intramuscular; IV: Intravenous; M. canariasense: *Mycobacterium canariasense*; R: Resistant; RAPMYCO: Rapid growing mycobacteria plate format; S: Sensitive.

## Competing interests

The authors declare that they have no competing interests.

## Authors’ contributions

DA, MN and RN participated in the surgical treatment and follow-up of the patient. DS and NV participated in the microbiological investigations. DA, NK and GA performed a literature review. DA wrote the article. NK and GA participated in the writing of article. All authors read and approved the final manuscript.

## Pre-publication history

The pre-publication history for this paper can be accessed here:

http://www.biomedcentral.com/1471-2334/14/238/prepub
